# The mediation effects of smartphone addiction, negative emotion and psychological resilience in the relationship between social support and sleep quality among medical students

**DOI:** 10.3389/fpsyt.2026.1839572

**Published:** 2026-06-10

**Authors:** Yanrong Zhang, Min Liu, Wenxin Yu, Zihang Gao, Dan Zhao, Wenjun Yan, Wei Wang, Xiuyin Gao, Qingzhi Wang

**Affiliations:** 1 Department of Community and Health Education, School of Public Health, Xuzhou Medical University, Xuzhou, China; 2 Center for Medical Statistics and Data Analysis, Xuzhou Medical University, Xuzhou, China

**Keywords:** medical students, negative emotion, psychological resilience, sleep quality, smartphone addiction, social support

## Abstract

**Purpose:**

Poor sleep quality and its serious consequences have become an urgent public health issue, but there is a lack of evidence in medical students. This study aimed to examine the multiple associations between sleep quality, social support, psychological resilience, smartphone addiction, and negative emotions among medical students, in order to provide a basis for targeted intervention measures.

**Methods:**

A cross-sectional study involving 716 medical students was conducted using a self-report questionnaire that assessed demographic characteristics, Perceived Social Support Scale, Connor-Davidson Resilience Scale-10, Mobile Phone Addiction Index, Depression Anxiety Stress Scales-21, and Pittsburgh Sleep Quality Index. Pearson correlation analysis and structural equation modeling (SEM) were performed to examine the association between social support, smartphone addiction, negative emotion, psychological resilience and sleep quality.

**Results:**

Correlation analysis showed that social support (r=-0.386, *p* < 0.01) and psychological resilience (r=-0.383, *p* < 0.01) were negatively correlated with poor sleep quality, and smartphone addiction (r=0.447, *p* < 0.01) and negative emotion (r=0.551, *p* < 0.01) were positively correlated with poor sleep quality. The SEM results indicated that social support exerts a direct positive effect on sleep quality and also an indirect effect on sleep quality via smartphone addiction and negative emotion, which serve as both separate and chain mediators. In contrast, psychological resilience has no direct impact on sleep quality, but it exerts an indirect effect on sleep quality through the same dual mediating pathways of smartphone addiction and negative emotion.

**Conclusion:**

This study clarifies the differential pathways through which social support and psychological resilience influence sleep quality via SEM analysis. These findings supplement the theoretical framework of sleep quality influencing factors, highlight the core mediating role of smartphone addiction and negative emotion, and provide targeted insights for formulating interventions to improve individuals’ sleep health.

## 1 Introduction

Sleep quality refers to individuals’ subjective self-perception of sleep, and is an important indicator for measuring physical and mental health (1). Poor sleep quality and other sleep related illnesses have become a prevalent public health concern among adolescents worldwide (2–4). A meta-analysis of 430,000 Chinese adolescents showed that the comorbidity rate of poor sleep quality with other problems reached 26% (5), and similar prevalence rates have been documented globally, including 24.6% among university students in the United States (6), and 42.8% in Germany (7). These convergent findings indicate that sleep problems transcend geographical and cultural boundaries, constituting a shared global health challenge among young populations. Meanwhile, a large amount of evidence has confirmed that poor sleep quality can trigger s series of adverse outcomes (8, 9), including decreased attention and cognitive function, obesity, anxiety, and depression. Critically, in some cases sleep related diseases have been considered as the important risk factors for elevated suicidal ideation and self-harm behaviors in adolescents (10, 11), further highlighting the severe public health burden of sleep problems. Given this, exploring correlated factors to sleep quality, and developing targeted prevention and intervention measures are of great practical significance for protecting young people’s health.

Existing researches have extensively explored the factors of sleep quality in psychological, social and behavior fields (4, 12–15). From the psychological dimension, two core protective factors have gained increasing attention: social support and psychological resilience. As a well-documented external protective factor, social support exerts a crucial protective effect on sleep quality (16–19). The Stress buffering hypothesis provides a solid theoretical foundation for this association, suggesting that adequate social support can reduce individuals stress perception, alleviate levels of anxiety and stress, and buffering their destructive effects on sleep quality (20). Correspondingly, psychological resilience as a vital intrinsic psychological protective resource (21), reflects individuals’ capacity to cope with adversity and stress. Numerous studies have shown that psychological resilience is closely correlated with sleep quality, as it helps individuals alleviate perceived external stress, regulate psychological states, and thereby maintain sleep quality (22, 23). However, it is notable that current evidence in psychological field remains with obvious population limitations and research gaps. Most relevant studies have concentrated on general adolescents or elderly patients, while insufficient attention has been paid to college student groups, especially medical students. And few studies have simultaneously incorporated social support and psychological resilience into one analytical system to compare their underlying mechanisms on sleep quality.

Globally, medical students are considered to be particularly susceptible to sleep related problems (24, 25), which is closely tied to the unique pressure context of medical education. Beyond heavy coursework, medical students face long-term academic competition, rigorous assessment mechanisms and clinical internship (26, 27). Such sustained high-intensity occupational exposure brings chronic psychological burden, forming a distinctive pathological stressor that differentiates medical students from peers in other majors. Researches showed that medical students commonly experience chronic stress, negative emotional accumulation and maladaptive behavioral coping (28, 29) and excessively rely on smartphones as a passive stress relief method (30, 31). Although the lack of sleep among medical students has attracted increasing attention, current evidence still lacks in-depth exploration of the mechanisms behind their sleep quality. In this high-risk population, further verification is needed to determine whether social support and psychological resilience can stabilize sleep quality, as well as the mediating relationship between behavioral and emotional risk factors.

As a prevalent behavioral addiction in the digital era, smartphone addiction has gradually evolved into a major environmental stressor threatening young people’s sleep health (32–34). Smartphone addiction disrupts sleep quality through multiple physiological and psychological pathways, characterized by compulsive craving, behavior avoidance, and low-efficient behavior (35). Previous researches have shown that adolescents face difficulties in using smartphones, and this uncontrollable dependence behavior seems to be closely related to sleep quality (32, 36, 37). From the perspective of behavioral addiction theory, the uncontrolled pre-bed smartphone use prolongs pre-sleep wakefulness, increase cognitive arousal level, and disrupts normal sleep rhythm. A systematic review on the relationship between digital media use and sleep outcomes among adolescents indicated that, the blue light and electromagnetic fields emitted by smartphones can alter brain activity, stimulate emotional dysregulation, enhance cognitive arousal, and thus affect sleep quality (38).

Negative emotion, such as anxiety, depression, and loneliness, is the typical manifestation of emotional dysfunction and a critical factor connecting stressors and health outcomes (20). Numerous studies have confirmed the close correlation between negative emotion and social support, psychological resilience, and smartphone addiction (13, 39, 40). Low levels of social support and psychological resilience tend to exacerbate smartphone addiction and negative emotion, which further lead to sleep quality. On this basis, it is reasonable to infer that negative emotion and smartphone addiction may be key variables in sleep quality, forming a continuous chain pathway between protective factors and sleep quality.

**Figure 1:**
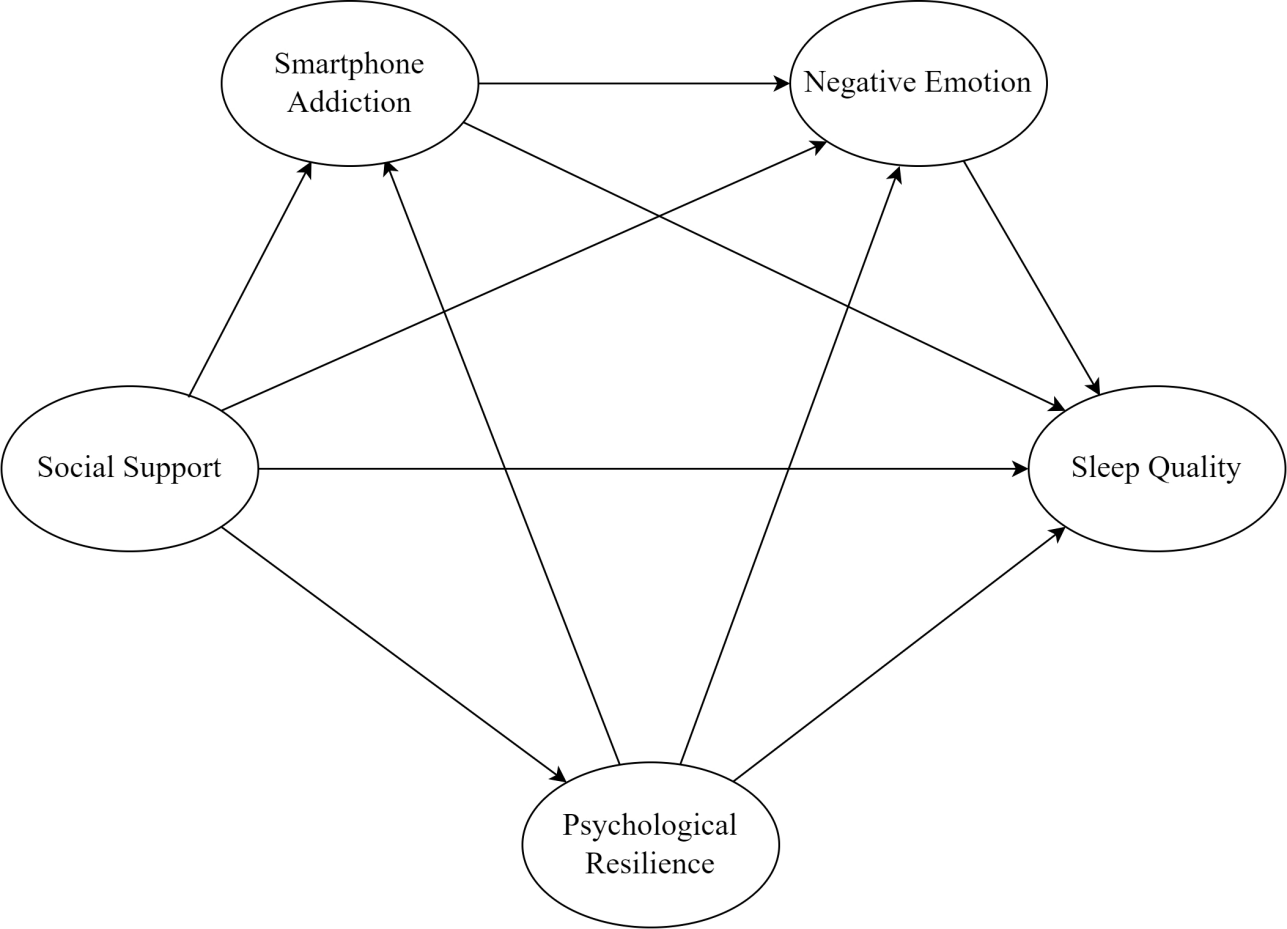
Hypothetical model.

This study constructed a comprehensive chained mediation framework, and incorporated social support, psychological resilience, smartphone addiction, negative emotion and sleep quality into one model (as shown in **Figure 1**), the research hypotheses are proposed as follows. Our design aimed to clarify the complex interactive relationships between protective factors, risk factors and sleep quality, and provided empirical evidence and targeted theoretical guidance for universities to carry out psychological intervention and smartphone use management. It is expected to improve medical students’ sleep quality, and promote their long-term physical and mental health and professional development.

H1: Smartphone addiction mediates the association between social support and sleep quality.

H2: Negative emotion mediates the association between social support and sleep quality.

H3: Smartphone addiction and negative emotion play a chained mediating role in the relationship between social support and sleep quality.

H4: Smartphone addiction mediates the association between psychological resilience and sleep quality.

H5: Negative emotion mediates the association between psychological resilience and sleep quality.

H6: Smartphone addiction and negative emotion play a chained mediating role in the relationship between psychological resilience and sleep quality.

H7: Psychological resilience mediates the association between social support and sleep quality.

## 2 Methods

### 2.1 Participants

A stratified cluster sampling method was employed to select medical students from Xuzhou Medical University to complete a questionnaire survey. After obtaining informed consent from students, a collective online survey was conducted during class, and participants voluntarily scanned the questionnaire access link. A total of 750 questionnaires were distributed, after excluding incomplete or invalid responses questionnaires, 716 valid questionnaires were obtained, with an effective recovery rate of 95.47%. This study was approved by the Ethics Committee of Xuzhou Medical University and adhered strictly to the principles of the Helsinki Declaration. All participants provided written informed consent after being fully informed of the research purpose, content, procedures, data usage and their rights and obligations.

Inclusion criteria: undergraduate medical students from freshman to fifth year who voluntarily participated after reading the study instructions. Exclusion criteria: (1) The questionnaire completion time less than two minutes; (2) those who have obvious contradictions in answering questions;(3) those who choose the same answer for all options.

### 2.2 Measures

#### 2.2.1 Social demographic characteristics

Social demographic characteristics about medical students, including gender, only child, residence, monthly living expenses, annual family income, academic ranking and self-rated health was collected.

#### 2.2.2 Social support

Medical students’ social support was measured using the Perceived Social Support Scale (PSSS), developed by Zimet et al (41), and adapted into Chinese by Jiang et al (42). The scale comprises 12 items across three dimensions: family support, friend support and other support. It employs a 7-point Likert scale (1=strongly disagree, 7=strongly agree), the total score ranges from 12 to 84, with a higher total score indicating a higher level of social support. The internal consistency of the scale in this study was good, with a Cronbach’s α coefficient of 0.938.

#### 2.2.3 Psychological resilience

The Connor-Davidson Resilience Scale-10 (CD-RISC-10) is a simplified version of the CD-RISC (43), adapted into Chinese by Wang et al (44). It assesses the dynamic process of successful adaptation in the face of adversity, trauma, risk, or other significant life stressors. The scale consists of 10 items, using a 5-point Likert scale (0=never, 4=almost always). The total score ranges from 0 to 40, with higher scores indicating higher levels of resilience. Both domestic and international scholars have conducted reliability and validity tests on the CD-RISC-10 (45, 46), consistently confirming its good reliability and validity. And the Cronbach’s alpha of this scale in this study was 0.926.

#### 2.2.4 Smartphone addiction

The assessment of smartphone addiction used the Mobile Phone Addiction Index (MPAI) (47) developed by Professor Leung Wing chi of The Chinese University of Hong Kong. This scale contains 17 items, divided into four dimensions: loss of control, withdrawal, avoidance, and low efficiency. Using the 5-point Likert rating system (ranging from “never” to “almost always”). The total score ranges from 0 to 68, and the higher the score, the higher the dependence on mobile phone. This scale has shown excellent reliability and validity in previous research (48–50). In this study, the Cronbach alpha coefficient was 0.900.

#### 2.2.5 Negative emotion

The Depression Anxiety Stress Scales-21(DASS-21) (51) was used to assess three negative emotion among medical students: anxiety, depression, and stress. The scale consists of three subscales, each containing 7 items, totaling 21 items. It uses a 4-point Likert scale (0=did not apply to me at all, 3=applied to me very much or most of the time”). The total score for each subscale ranges from 0 to 21. Higher scores indicate higher levels of anxiety, depression, and stress. The DASS-21 has been proven to be a reliable and valid measure of mental health (52, 53). The Cronbach’s alpha of this scale in this study was 0.950.

#### 2.2.6 Sleep quality

The Pittsburgh Sleep Quality Index (PSQI), developed by Buysse Citation (54), was to assess medical students’ sleep quality. The scale evaluates sleep quality over the previous month through 18 items grouped into seven components: subjective sleep quality, sleep latency, sleep duration, sleep efficiency, sleep disturbances, use of sleeping medication, and daytime dysfunction. Each component is scored on a scale from 0 to 3, resulting in a total score range of 0 to 21. The higher the score, the poorer the sleep quality. This measurement tool has been extensively validated and widely utilized in Chinese population (13, 55). In this study, the scale’s Cronbach’s alpha was 0.740, demonstrating good internal consistency.

### 2.3 Statistical analysis

All data were analyzed using SPSS 24.0 and Amos 24.0, with a significance level of *P* < 0.05 (two sided) considered statistically significant. Quantitative data were presented as mean ± standard deviation, and Count data was described in frequency and percentage. T-tests or Analysis of Variance (ANOVA) were conducted to explore the differences between social demographic characteristics and sleep quality.

Pearson correlation analysis was used to clarify the correlations between social support, psychological resilience, smartphone addiction, negative emotion and sleep quality.

**Figure 2:**
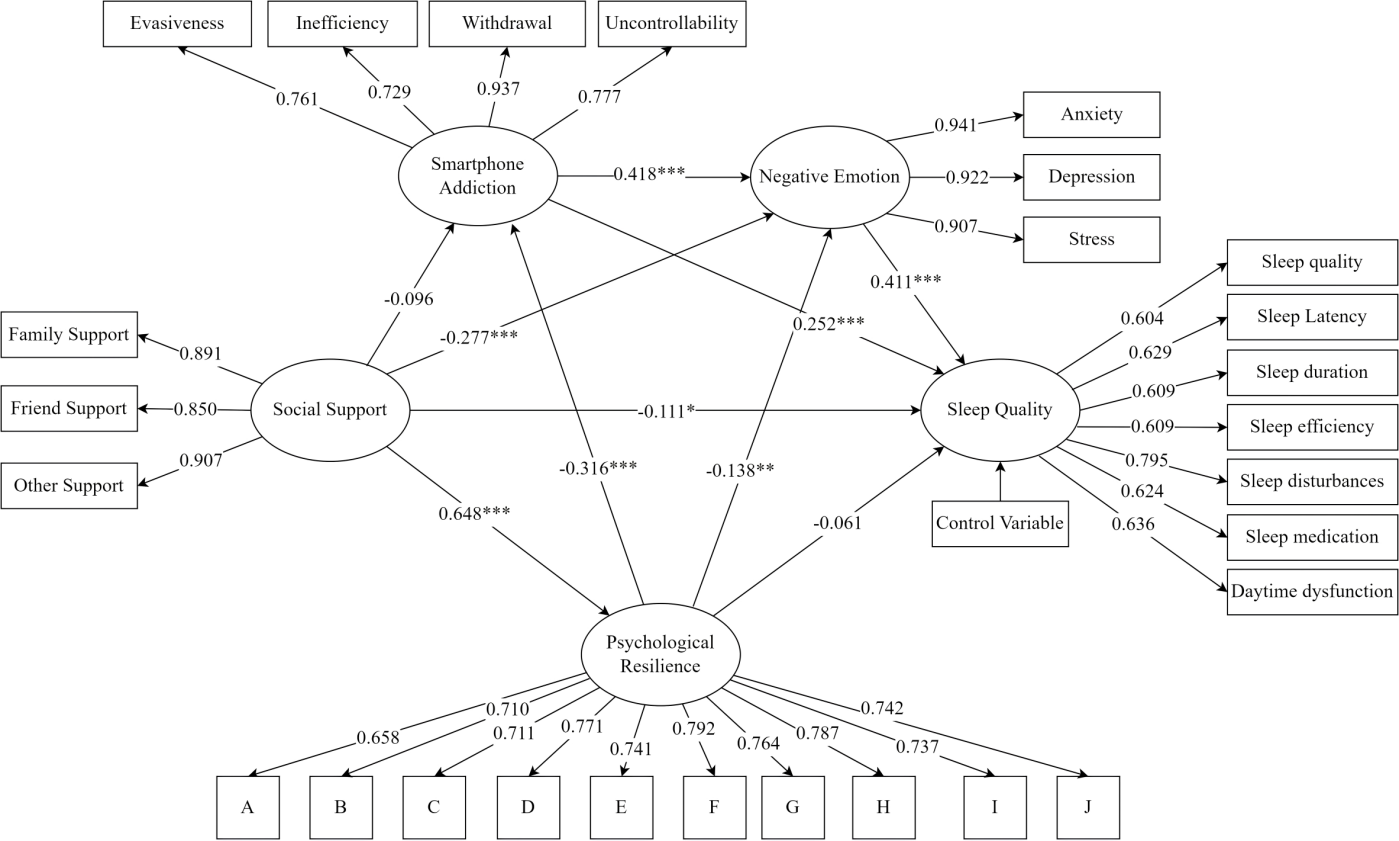
Mediation model of smartphone addiction, negative emotion and psychological resilience between social support and sleep quality. **(A–J)** represent ten constructs of psychological resilience. Control variables included grade, monthly living expenses, annual family income, and self-rated health. *P < 0.05, **P < 0.01, ***P < 0.001.

The structural equation model was established using Amos 24.0 software, and the maximum likelihood estimation method was employed to assess the hypothesized model. In this study, a series of indices were used to assess model fit, including the chi-square to degrees of freedom ratio (CMIN/DF), goodness-of-fit index (GFI), adjusted goodness-of-fit index (AGFI), comparative fit index (CFI), incremental fit index (IFI), standardized root-mean-square residuals (SRMR), and root-mean-square error of approximation (RMSEA). A model is considered to have a satisfactory fit if it shows indices above 0.90 for GFI, AGFI, CFI and IFI, and below 0.08 for SRMR and RMSEA (56, 57). Non-significant hypothesized paths (p >.05) were removed sequentially, beginning with the least theoretically central path, and model fit was re-evaluated after each removal. When the initial model exhibited inadequate fit, we examined modification indices (MIs) to identify potential sources of model misspecification. Residual correlations were added between specific observed variables only when (a) the MI exceeded 10, (b) the expected parameter change was substantively meaningful, and (c) there was a clear theoretical or empirical justification (e.g., similar item wording, shared method variance, or conceptual overlap). No non-hypothesized structural paths were added, and any path modifications were guided by theoretical considerations rather than data-driven exploration alone. The final model specification is reported in **Figure 2**.

Bootstrap resampling with 5,000 iterations was conducted to estimate the total, direct, and indirect effects within the hypothesized mediation model. Bias-corrected and accelerated (BCa) 95% confidence intervals (CIs) were computed for all indirect effects. An indirect effect was deemed statistically significant if its 95%CI did not contain zero. This approach was chosen because BCa intervals provide more accurate coverage than standard percentile intervals, particularly when the sampling distribution of indirect effects is non-normal.

## 3 Results

### 3.1 Common method biases test

To evaluate the potential influence of common method biases in cross-sectional design, we conducted Harman’s single-factor test. The results indicated that through principal component factor analysis, we extracted 9 feature values greater than 1, the variance explained by the first factor is 30.156%, which was below the critical threshold of 40%, indicating that common method bias was not a serious issue in this study.

### 3.2 Characteristics and comparisons of sleep quality

**Table 1** displays the participants’ characteristics and comparisons of sleep quality. In this study, 50.84% of the participants were male, 59.36% were freshman year students, the majority (60.34%) came from urban areas and more than half of them were not only child (56.28%). Additionally, 33.10% had a monthly living expense of 1501–2000 yuan, 39.39% had a family annual income above 100,000 yuan, and 32.68% of participants’ academic performance ranked in 10%-30% in the previous year. Nearly half of the participants reported self-rated health as average (50.00%). The results showed that, there was a significant statistical significance between grade (F = 32.831, *p* < 0.001), monthly living expenses (F = 18.005, *p* < 0.001), annual family income (F = 6.389, *p* < 0.001), self-rated health (F = 29.313, *p* < 0.001) and sleep quality.

**Table 1:** Characteristics and comparisons of sleep quality among medical students (N = 716).

Variables	n (%)	Sleep quality ( $\bar{X}$ ± *SD*)	T/F	*p*
**Gender**			1.132	0.288
Male	364 (50.84)	0.90 ± 0.52		
Female	352 (49.16)	0.94 ± 0.49		
**Grade**			32.831	<0.001
Freshman year	425 (59.36)	0.77 ± 0.45		
Sophomore year	73 (10.20)	1.14 ± 0.52		
Junior year	88 (12.29)	1.23 ± 0.49		
Senior year	83 (11.59)	1.20 ± 0.44		
Fifth year	47 (6.56)	0.85 ± 0.49		
**Residence**			0.870	0.351
Urban	432 (60.34)	0.91 ± 0.50		
Rural	284 (39.66)	0.94 ± 0.51		
**Only child**			1.376	0.241
Yes	313 (43.72)	0.95 ± 0.53		
No	403 (56.28)	0.90 ± 0.48		
**Monthly living expenses** **(yuan)**			18.005	<0.001
<800	52 (7.26)	1.33 ± 0.47		
800-1000	98 (13.69)	1.13 ± 0.48		
1001-1500	192 (26.82)	0.89 ± 0.45		
1501-2000	237 (33.10)	0.81 ± 0.47		
>2000	137 (19.13)	0.85 ± 0.53		
**Annual family income** **(yuan)**			6.389	<0.001
<30,000	102 (14.25)	1.07 ± 0.53		
30,000-60,000	149 (20.81)	0.97 ± 0.50		
60,000-100,000	183 (25.56)	0.94 ± 0.04		
>100,000	282 (39.39)	0.84 ± 0.49		
**Academic ranking**			2.106	0.078
Top 10%	121 (16.90)	0.97 ± 0.55		
10%-30%	234 (32.68)	0.87 ± 0.48		
30%-50%	157 (21.93)	0.89 ± 0.51		
50%-70%	127 (17.74)	1.00 ± 0.51		
Last 30%	77 (10.75)	0.96 ± 0.45		
**Self-rated health**			29.313	<0.001
Bad	113 (15.78)	1.23 ± 0.43		
Average	358 (50.00)	0.91 ± 0.48		
Good	245 (34.22)	0.81 ± 0.52		

Bold text indicates category headings for demographic variables.

### 3.3 Correlation between variables

**Table 2** presents the preliminary Pearson correlations among social support, smartphone addiction, psychological resilience, negative emotion, and sleep quality. These bivariate correlations are reported as a data-quality screening step prior to the main SEM analysis, not as a substitute for the model-estimated structural paths. As shown in **Table 2**, there was a negative correlation between social support and sleep quality (r = −0.386, p < 0.01), a negative correlation between psychological resilience and sleep quality (r = −0.383, p < 0.01), a positive correlation between smartphone addiction and sleep quality (r = 0.447, p < 0.01), and a positive correlation between negative emotion and sleep quality (r = 0.551, p < 0.01). All associations were in the expected directions, supporting the appropriateness of the data for subsequent SEM estimation.

**Table 2:** Correlations between sleep quality, social support, psychological resilience, smartphone addiction, and negative emotion.

Variables	$\bar{X}$ ± *SD*	1	2	3	4	5
1Sleep quality	0.92 ± 0.50	1				
2Social support	3.41 ± 0.84	-0.386**	1			
3Psychological resilience	2.34 ± 0.81	-0.383**	0.594**	1		
4Smartphone addiction	2.38 ± 0.77	0.447**	-0.262**	-0.330**	1	
5Negative emotion	0.89 ± 0.63	0.551**	-0.456**	-0.436**	0.497**	1

**indicates *P* < 0.01.

### 3.4 Mediating effect analysis

Based on the research hypothesis, we constructed a structural equation model on the relationship between sleep quality, social support, psychological resilience, smartphone addiction and negative emotion (**Figure 2**). The initial model results showed that GFI and AGFI did not meet the required standards. Further modifications were made to the model by establishing the correlation between the residuals of psychological resilience dimensions. The fitting index of the final revised model met the established goodness of fit standard (**Table 3**), indicating that the hypothesized model fitted well with the empirical data model.

**Table 3:** Fitness indexes of the model.

Variables	CMIN/DF	GFI	AGFI	IFI	CFI	RMSEA	SRMR
Reference	<3.0	>0.9	>0.9	>0.9	>0.9	<0.08	<0.08
Initial	3.172	0.899	0.879	0.937	0.937	0.055	0.071
Revised	2.752	0.914	0.901	0.950	0.950	0.049	0.070

CMIN/DF, degrees of freedom; GFI, goodness-of-fit index; AGFI, adjusted goodness-of-fit index; IFI, incremental fit index; CFI, comparative fit index; SRMR, standardized root-mean-square-residual; RMSEA, root-mean-square-error-of- approximation.

Using bias-corrected percentile bootstrap procedures (5,000 resamples), we examined the indirect pathways specified in the hypothesized model. As presented in **Table 4**, several indirect effects were statistically significant, though their magnitudes varied considerably. Social support was associated with sleep quality through smartphone addiction (β = −0.023, 95% CI [−0.051, −0.002]), with this pathway accounting for 8.7% of the total effect, consistent with H1. A larger mediated association was observed through negative emotion (β = −0.114, 95% CI [−0.175, −0.068]), accounting for 32.4% of the total effect, consistent with H2. The chained pathway via smartphone addiction and negative emotion was also significant (β = −0.016, 95% CI [−0.031, −0.002]), though it accounted for a modest 6.2% of the total effect, consistent with H3. The total association between social support and sleep quality was β = −0.111 (95% CI [−0.215, −0.024]), with the mediators collectively explaining 42.1% of this association. Psychological resilience was associated with sleep quality through smartphone addiction (β = −0.080, 95% CI [−0.125, −0.055]), accounting for 35.9% of the total association, consistent with H4. The pathway via negative emotion was also significant (β = −0.057, 95% CI [−0.095, −0.010]), accounting for 25.7% of the total effect, consistent with H5. The chained pathway via both mediators was significant (β = −0.024, 95% CI [−0.042, −0.005]), accounting for 10.7% of the total effect, consistent with H6. The total association between psychological resilience and sleep quality was β = −0.061 (95% CI [−0.150, 0.023]), with the mediators collectively explaining 27.7% of this association. Notably, psychological resilience was not significantly associated with sleep quality when positioned as a mediator between social support and sleep quality (β = −0.040, 95% CI [−0.103, 0.014]), which was inconsistent with H7.

**Table 4:** The standardized total, direct, and indirect effects of social support on sleep quality.

Hypothesis	Model path	*β*	95%CI	Percent (%)	Result
	SS→SQ	-0.111***	(-0.215, -0.024)	42.1
H1	SS→SP→SQ	-0.023***	(-0.051, -0.002)	8.7	Conformed
H2	SS→NE→SQ	-0.114***	(-0.175, -0.068)	43.3	Conformed
H3	SS→SP→NE→SQ	-0.016***	(-0.031, -0.002)	5.9	Conformed
	PR→SQ	-0.061	(-0.150, 0.023)	27.7	
H4	PR→SP→SQ	-0.080***	(-0.125, -0.055)	35.9	Conformed
H5	PR→NE→SQ	-0.057***	(-0.095, -0.010)	25.7	Conformed
H6	PR→SP→NE→SQ	-0.024***	(-0.042, -0.005)	10.7	Conformed
H7	SS→P→SQ	-0.040	(-0.103, 0.014)		Not conformed

***P < 0.001, SE, standard error; CI, confidence interval; SQ, sleep quality; SS, social support; SP, smartphone addiction; PR, psychological resilience; NE, negative emotion.

## 4 Discussion

This study tested multiple hypotheses aimed at exploring the multi-path associations between sleep quality and social support, psychological resilience, smartphone addiction, and negative emotion among medical students. The results indicated that almost all hypotheses were supported, except for the mediating role of psychological resilience between social support and sleep quality.

### 4.1 Mediating roles of smartphone addiction and negative emotion

Our study found that both smartphone addiction and negative emotion played the mediating roles between social support and sleep quality, covering independent and chained associative pathways. Notably, the indirect effect via smartphone addiction and the chained effect were relatively modest in magnitude (accounting for 8.7% and 6.2% of the total association, respectively), suggesting that the correlation between social support and sleep quality was most strongly reflected through negative emotion. Our finding that insufficient social support was associated with elevated negative emotion among medical students, aligns with prior research on adolescent populations (58–60), supporting the generalizability of the social support–negative emotion link across young adults. From the perspective of the stress buffering hypothesis (20), when the social support medical students received, such as peer assistance, teacher care, and family emotional support are insufficient, negative experiences are difficult to effectively alleviate, which can then transform into persistent anxiety and fatigue. These negative emotions are the direct causes that affect sleep quality, leading to problems such as difficulty falling asleep and insufficient sleep. And in the chain mediation pathway, negative emotion served as the ultimate transmission node, amplifying the negative effect of social support and smartphone addiction. Even if insufficient social support indirectly correlated sleep quality by smartphone addiction, this behavior pathway contributed only a small explanatory share. Ultimately, the association between inadequate social support and sleep quality was amplified through the accumulation of negative emotion, which served as the most critical indirect factor in the relationship between social support and sleep quality.

Differing from the associative pathway between social support and sleep quality, our study found that smartphone addiction exhibited a more prominent intermediate association between psychological resilience and sleep quality. This correlation can be explained by the stress coping theory (61). People with low levels of psychological resilience often lack adaptive stress management strategies when facing long-term stress, which is highly consistent with the educational intensity and occupational stress background of medical students (28, 29). Research has found that smartphone addiction among medical students is a stress maladaptive avoidance behavior (62), including using prolonged online socializing and mobile games to escape the psychological discomfort of academic anxiety. This type of smartphone addiction relies on directly invading the sleep time of medical students, disrupting their sleep rhythm (63, 64). More importantly, poor sleep quality may also in turn prolong screen time and strengthen smartphone addiction, forming a vicious cycle (64), and therefore smartphone addiction had a more direct and strong correlation with sleep quality. However, the role of negative emotion in the association between psychological resilience and sleep quality was relatively low, which may be because low levels of psychological resilience did not directly trigger negative emotion among medical students. The relationship between low-level psychological resilience and smartphone dependence was more closely related, and negative emotion may be a secondary accompanying factor in this association pathway.

These differential results provided relevant evidence and inspiration for us to improve the sleep quality of medical students. Different from the common one size fits all intervention plan, pre-evaluating the levels of social support and psychological resilience of medical students and developing strategies that focus on smartphone addiction management or negative emotion counseling based on individual levels may be more beneficial for optimizing the sleep quality of medical students.

### 4.2 Mediating roles of psychological resilience

Psychological resilience did not demonstrate a significant mediating role between social support and sleep quality. This null finding contrasts with prior research (13, 65–67), and several factors may contribute to this discrepancy. First, medical students face unique chronic stressors (68, 69) that may alter the typical psychological resilience–sleep quality association observed in general populations. Second, the residual correlations added between psychological resilience subdimensions during model modification indicate substantial shared variance among these indicators, which may have attenuated the latent variable’s independent predictive power. Meanwhile, the dimensional setting of the psychological resilience scale in our study may be another critical reason. The measurement of psychological resilience in this study was mainly focused on stress resistance and environmental adaptation, and its association with sleep quality was naturally difficult to capture. In addition, the revision of establishing residual correlations between sub dimensions of psychological resilience also provided a reasonable supplementary explanation for this insignificant mediating association. Residual correlation reflects measurement errors and homogeneity attributes within the same underlying structure, indicating that the internal structural homogeneity of psychological resilience dimensions was prominent in our study sample. The strong internal correlation between sub dimensions may weaken the independent explanatory power of psychological resilience as an overall latent variable, further limiting its mediating association between social support and sleep quality.

### 4.3 Limitations

Firstly, as a cross-sectional study, this study cannot explore the long-term effects and causal relationships between variables. Future research should consider conducting prospective cohort studies. Secondly, relying on self-reported data may lead to common methodological biases. Thirdly, our study only examined the mediating effects of variables such as smartphone addiction, and other possible mediating factors need to be further explored. Finally, we did not consider the existence of confounding factors in our study. For example, the epidemic caused by novel coronavirus in 2019 seems to be related to the negative emotion of medical students (70, 71).

## 5 Conclusion

This study examined the associations among social support, psychological resilience, smartphone addiction, negative emotion, and sleep quality among medical students, and explored hypothesized mediating pathways within a cross-sectional framework. The results indicated that smartphone addiction and negative emotion were each associated with the social support–sleep quality and psychological resilience–sleep quality relationships, both independently and in sequence. Negative emotion exhibited a relatively stronger associative role in the social support–sleep quality link, whereas smartphone addiction showed a more prominent associative role in the psychological resilience–sleep quality link. These findings provide preliminary correlational evidence that, if replicated in longitudinal or experimental designs, may inform the development of hypothesis-driven intervention approaches for medical students. Specifically, future research could examine whether emotion-focused strategies are more relevant for individuals with low social support, and whether behavioral regulation strategies are more relevant for individuals with low psychological resilience.

## Data Availability

The original contributions presented in the study are included in the article/supplementary material. Further inquiries can be directed to the corresponding authors.
